# PB.3. Detection of lymph node metastases in newly diagnosed breast cancer patients with axillary ultrasonography in combination with the Memorial Sloan Kettering Cancer Centre normogram as a prediction tool

**DOI:** 10.1186/bcr3733

**Published:** 2014-11-03

**Authors:** CS Low, S Meraj, M Sreenivas

**Affiliations:** 1 University Hospitals Coventry and Warwickshire NHS Trust, Coventry, UK

## Introduction

Current axillary lymph node assessment criteria by ultrasonography (US) depends on cortical thickness >3 mm, focal eccentric cortical thickness and lobulated cortex. Combination with a prediction tool may define better patient management strategies.

## Methods

Newly diagnosed breast cancer patients within a 1-year period were randomly selected retrospectively. Inclusion criteria include patients with final histological correlation following resection. The Memorial Sloan Kettering Cancer Centre breast cancer normogram has been utilised. Axillary nodal US findings and histological correlation from fine needle aspiration cytology (FNAC)/core biopsies were obtained.

## Results

A total of 70/160 randomly selected patients fulfilled the inclusion criteria.

In total, 28/70 patients were positive for axillary nodal metastases. A total 15/28 patients underwent FNAC/core biopsy and 11 were positive. In the remaining 13 patients, nodal metastases were detected by sentinel lymph node biopsy (SLNB) in 10 patients and initial axillary node clearance (ANC) in three patients. These 13 patients had a probability range of 26 to 91% (mean 59%). The overall probability range in all 28 patients is 6 to 97% (mean 64%). Forty-two patients did not have lymph node metastases. One patient did not have axillary US assessment. In total, 10/41 patients had abnormal nodes on US that were negative on FNAC/core biopsy. All 42 patients had surgical lymph node assessment (38 had SLNB, four had ANC). The overall probability range is 9 to 80% (mean 36%).

## Conclusion

The detection of axillary nodal metastases with US remains low but combination with the normogram prediction tool may be helpful to determine patients with high probability to have repeated US assessment and sampling of normal-looking nodes to increase the detection rate.

